# The Efficacy of *Camelina sativa* Defatted Seed Meal against Colitis-Induced Persistent Visceral Hypersensitivity: The Relevance of PPAR α Receptor Activation in Pain Relief

**DOI:** 10.3390/nu14153137

**Published:** 2022-07-29

**Authors:** Elena Lucarini, Laura Micheli, Eleonora Pagnotta, Alessandra Toti, Valentina Ferrara, Clara Ciampi, Francesco Margiotta, Alma Martelli, Lara Testai, Vincenzo Calderone, Roberto Matteo, Serafino Suriano, Antonio Troccoli, Nicola Pecchioni, Clementina Manera, Lorenzo Di Cesare Mannelli, Carla Ghelardini

**Affiliations:** 1 Department of Neuroscience, Psychology, Drug Research, and Child Health—NEUROFARBA—Pharmacology and Toxicology Section, University of Florence, 50139 Florence, Italy; elena.lucarini@unifi.it (E.L.); laura.micheli@unifi.it (L.M.); alessandra.toti@unifi.it (A.T.); valentina.ferrara@unifi.it (V.F.); clara.ciampi@stud.unifi.it (C.C.); francesco.margiotta@unifi.it (F.M.); carla.ghelardini@unifi.it (C.G.); 2 CREA—Council for Agricultural Research and Economics, Research Centre for Cereal and Industrial Crops, 40128 Bologna, Italy; eleonora.pagnotta@crea.gov.it (E.P.); roberto.matteo@crea.gov.it (R.M.); 3 Department of Pharmacy, University of Pisa, 56126 Pisa, Italy; alma.martelli@unipi.it (A.M.); lara.testai@farm.unipi.it (L.T.); vincenzo.calderone@unipi.it (V.C.); clementina.manera@unipi.it (C.M.); 4 Interdepartmental Research Centre Nutraceuticals and Food for Health—NUTRAFOOD, University of Pisa, 56126 Pisa, Italy; 5 Interdepartmental Research Centre of Ageing Biology and Pathology, University of Pisa, 56126 Pisa, Italy; 6 CREA—Council for Agricultural Research and Economics, Research Centre for Cereal and Industrial Crops, 71122 Foggia, Italy; serafino.suriano@crea.gov.it (S.S.); antonio.troccoli@crea.gov.it (A.T.); nicola.pecchioni@crea.gov.it (N.P.)

**Keywords:** inflammatory bowel diseases, mast cell, enteric nervous system, PPAR α receptor, 2,4-dinitrobenzenesulfonic acid, visceral pain, *Camelina sativa*

## Abstract

*Brassicaceae* are natural sources of bioactive compounds able to promote gut health. Belonging to this plant family, *Camelina sativa* is an ancient oil crop rich in glucosinolates, polyunsaturated fatty acids, and antioxidants that is attracting renewed attention for its nutraceutical potential. This work aimed at investigating the therapeutic effects of a defatted seed meal (DSM) of *Camelina sativa* on the colon damage and the persistent visceral hypersensitivity associated with colitis in rats. Inflammation was induced by the intrarectal injection of 2,4-dinitrobenzenesulfonic acid (DNBS). The acute administration of *Camelina sativa* DSM (0.1–1 g kg^−1^) showed a dose-dependent pain-relieving effect in DNBS-treated rats. The efficacy of the meal was slightly enhanced after bioactivation with myrosinase, which increased isothiocyanate availability, and drastically decreased by pre-treating the animals with the selective peroxisome proliferator-activated receptor alpha (PPAR α) receptor antagonist GW6471. Repeated treatments with *Camelina sativa* DSM (1 g kg^−1^) meal counteracted the development, as well as the persistence, of visceral hyperalgesia in DNBS-treated animals by reducing the intestinal inflammatory damage and preventing enteric neuron damage. In conclusion, *Camelina sativa* meal might be employed as a nutraceutical tool to manage persistent abdominal pain in patients and to promote gut healing.

## 1. Introduction

Persistent or recurrent abdominal pain is a common symptom of gastrointestinal diseases [1]. Gut inflammation represents a major risk factor for the development of the persistent visceral hypersensitivity that underlies the onset of pain and altered motility and that often persists after the resolution of intestinal damage [2]. It is particularly evident in patients affected by inflammatory bowel diseases (IBDs), who continue suffering from abdominal pain and discomfort even after achieving clinical remission, developing a post-inflammatory irritable bowel syndrome (PI-IBS) [2,3].

Unfortunately, most of the classical anti-inflammatory drugs and analgesics show low efficacy for visceral pain and often display gastrointestinal side effects that impede their long-term employment in patients [4,5]. This opened the way for the investigation of novel therapeutic strategies, including psychotherapeutic and nutritional approaches [5,6]. Many plant-derived extracts have also been investigated with the aim of combining different mechanisms of action to enhance the efficacy on both gut symptoms like pain and altered motility and extra-intestinal symptoms like depression and anxiety associated with IBDs [5,7].

Visceral hyperalgesia resulting from colitis has a complex nature with inflammatory, neuropathic, and immune components [1,8]. The broad range of beneficial effects shown by *Brassicaceae* constituents, such as the glucosinolates (GSLs) [9,10], raised our interest in studying the potential of employing these plants in the therapy for gastrointestinal pain. Indeed, the main derivatives of GSL hydrolysis, isothiocyanates (ITCs), have been reported to modulate both inflammatory processes and oxidative stress [10,11], two factors contributing to the development of chronic pain in patients [3,6,12,13]. ITCs also display a prebiotic activity [12,13,14] that is highly relevant in the context of gut pain [15,16,17]. Notably, ITCs are effective against a wide range of inflammatory and neuropathic pain conditions by the slow release of H_2_S *in vivo* [11,18,19], followed by the activation of Kv7 potassium channels [18,20,21], which are involved in the transmission of painful stimuli [19,22] also from the viscera [23,24,25].

Among the less investigated *Brassicaceae* plants, there is *Camelina sativa*, also known as “false flax” and “gold of pleasure”, which is an ancient oil crop grown mainly in European countries [26,27,28]. This plant attracted much attention for producing functional food due to its high content of *n*-3 fatty acids [27,28]. On the other hand, camelina seeds are a source of GSLs like glucoarabin and glucocamelinin [29,30,31], though the *in vivo* biological effects of these phytochemicals are still largely unknown. Recent evidence from the literature showed that *Camelina sativa* seed alcoholic extracts can ameliorate cognitive performance, as well as mood, and exert an antioxidant effect in both the brain and gut in a mice model of IBS [32], though the effect on visceral pain was not investigated.

The aim of the present work was to investigate the anti-hyperalgesic and protective efficacy on the gut of administering a defatted seed meal (DSM) of *Camelina sativa* in a rodent model of colitis induced by the intrarectal instillation of DNBS [8,33]. The mechanisms of action of the *Camelina sativa* constituents in this context were also explored.

## 2. Materials and Methods

### 2.1. Camelina Sativa Defatted Seed Meal Production and Characterization

*Camelina sativa* var. Italia seeds belong to the brassica seed collection of CREA-CI (Bologna) [34]. *Camelina sativa* seeds were defatted using a continuous seed crusher (Bracco Company model Elle.Gi type 0.90) in a temperature-controlled procedure (temperature < 70 °C). *Camelina sativa* DSM was characterized by moisture, proteins, residual oil content, fatty acid composition, and GSLs using standard procedures as previously described [20]. GSL detection was performed after desulfation by means of sulfatase (200 μL, 0.35 U mL^−1^), purified from *Helix pomatia* (Merk Life Science S.r.l., Sigma Aldrich-solution, Milan, Italy) according to ISO 9167-1 (EN ISO 9167:2019). The desulphated GSLs were monitored at 229 nm and identified with comparison to literature retention times [35]; their amounts were estimated using purified sinigrin as an internal standard and 1 as the response factor in the absence of available relative response factors for the GSLs from the *Camelina sativa* seeds. Soluble and insoluble phenolic acids and flavonoids were extracted and analyzed under HPLC–UV at 280 and 320 nm wavelengths, following the procedures described in [36]. Compounds were identified based on their retention times and confirmed by their UV spectra in comparison with standards (gallic acid, protocatechuic acid, *p*-hydroxybenzoic acid, vanillic acid, syringic acid, vanillin, caffeic acid, *p*-coumaric acid, sinapic acid, trans-cinnamic acid, luteolin, vitexin, apigenin, naringenin, rutin, quercetin-3 glucoside). External calibration curves were prepared for each standard in a concentration range of 2.5–40 mg L^−1^. The amount of the compounds was quantified by comparing their peak areas with those of standard curves and finally expressed in µg g^−1^ of DSM.

### 2.2. Animals

Three-month-old male Sprague-Dawley rats (Envigo, Varese, Italy) with an initial average weight of 220–250 g were used for the experiments. Four animals per cage (size 26 cm × 41 cm) were housed in CeSAL (Centro Stabulazione Animali da Laboratorio, University of Florence, Florece, Italy) and used at least 1 week after their arrival. All animals were maintained on a 12-h light/dark cycle in a temperature-controlled environment (23 ± 1 °C) with access to food and water ad libitum. All animal manipulations were carried out according to Directive 2010/63/EU of the European Parliament and of the European Union council (22 September 2010) on the protection of animals used for scientific purposes. The ethical pol-icy of the University of Florence complies with the Guide for the Care and Use of Laboratory Animals of the US National Institutes of Health (NIH Publication No. 85-23, revised 1996; University of Florence assurance number: A5278-01). Formal approval to conduct the described experiments was obtained from the Animal Subjects Review Board of the University of Florence (543/2017-PR). Experiments involving animals have been reported according to ARRIVE guidelines [37]. All efforts were made to minimize animal suffering and to reduce the number of animals used.

### 2.3. Induction of Colitis

Colitis was induced as previously described [8]. Briefly, animals were anesthetized lightly with isoflurane (2%) and 30 mg of DNBS (Sigma-Aldrich, Milan, Italy) in 0.25 mL of 50% ethanol was administered intrarectally (8 cm proximal to the anus) through a polyethylene PE-60 catheter. As a vehicle group, some rats received 0.25 mL of saline solution.

### 2.4. Treatments

*Camelina sativa* DSM and myrosinase were produced according to the method described above. Compounds were acutely administered as follows. The doses of *Camelina sativa* DSM (0.1–1 g kg^−1^ po) were chosen based on previous evidence [25]. *Camelina sativa* DSM was bioactivated by adding 30 μL mL^−1^ of myrosinase (32 U mL^−1^) 15 min before the administration. A group of animals was administered *Camelina sativa* DSM (1 g kg^−1^) in a mixture with glutathione 65 μmol kg^−1^ (20 mg kg^−1^; GSSG; Sigma-Aldrich, Milan, Italy). The Kv7 potassium channel blocker XE991 (Bio-Techne, Milan, Italy; 1 mg kg^−1^), the PPAR-Ƴ receptor antagonist G3335 (Cayman Chemical, Vinci Biochem, Florence, Italy; 1 mg kg^−1^ in 5% DMSO + 5% TWEEN20), the PPAR-α receptor antagonist GW6471 (Bio-Techne, Milan, Italy; 2 mg kg^−1^ in 5% EtOH), the CB2 receptor antagonist MC21 (10 mg kg^−1^ in 5% DMSO + 5% TWEEN20) [38], or the CB1 receptor antagonist SR141716 (Bio-Techne, Milan, Italy; 10 mg kg^−1^ in 5% DMSO + 5% TWEEN20) were dissolved in saline solution and intraperitoneally administered 15 min before *Camelina sativa* DSM oral administration. All behavioral tests were carried out 30 min after the injection of *Camelina sativa* DSM.

Repeated oral administrations of *Camelina sativa* DSM (1 g kg^−1^) were carried out daily in rats, starting from the day of the DNBS injection and continuing for 14 days. Behavioral tests were performed 8 and 15 days after DNBS injection, 24 h after the last treatment.

### 2.5. Assessment of Visceral Sensitivity by Abdominal Withdrawal Reflex to Colorectal Distension

Visceral pain was measured by assigning a semi-quantitative score (0 to 4) to the abdominal withdrawal reflex (AWR) of animals in response to colorectal distension, as described previously [2,5]. Colorectal distension was performed by inflating a 4.5 cm balloon placed into the colon of animals with increasing volumes of water (0.5, 1, 2, 3 mL). Five minutes was the time elapsed between two consecutive distensions.

### 2.6. Histological Analysis of Colon

The presence of colon damage was investigated in accordance with the methods used in previous studies [6] by analysis ex vivo. Different parameters (adhesions between colon and other intra-abdominal organs; appearance of faeces; thickness of the tissue; hyperaemia and mucosal damage) were evaluated by the observation of the harvested colon to assign a macroscopic damage score. For the histological analysis, the colon was fixed in 4% paraformaldehyde for 24 h, dehydrated in alcohol, included in paraffin, and cut into 5 μm sections. Microscopic evaluations of colon damage (mucosal architecture loss, cellular infiltrate, muscle thickening, crypt abscess and goblet cell depletion) were carried out on haematoxy-lin/eosin-stained sections. The infiltration of mast cells (MCs) was investigated on colon sections stained with GIEMSA (Sigma-Aldrich, Milan, Italy) [7]. Digitalized images were collected by a Leica DMRB light microscope equipped with a DFC480 digital camera (40× magnification; Leica Microsystems, Wetzlar, Germany). The quantitative analysis was carried out by two blind investigators with the software ImageJ. For each animal, it was measured the cellular density (cell number/respective arbitrary field) of five independent arbitrary optical fields (0.1 mm^2^) collected from the submucosa.

### 2.7. Immunohistochemistry

For immunoreactions, tissue was cut into 5 µm slices and dried on glass slides prior to deparaffinization with xylol and rehydration in a descending alcohol series (100, 95, 75 and 50%). Tissues were rinsed three times (5 min each) in PBS containing 0.1% Triton X-100 (T-PBS) followed by a 1 h incubation in blocking solution (containing 0.1% Triton X-100, and 5% bovine serum albumin in 1X PBS) at room temperature. The slices were incubated overnight at 4 °C with a mouse anti-UCH-L1/PGP9.5 (Novus Biologicals-31A3, Bio-Techne Ltd., Abingdon, UK), diluted 1:500 in T-PBS/5% BSA (Sigma-Aldrich, Milan, Italy) and a rabbit anti-glial fibrillary acidic protein (GFAP, DAKO-Z0334, Agilent Technologies Italia, Milan, Italy), diluted 1:500 in PBS/5% BSA (Sigma-Aldrich, Milan, Italy). The following day, slides were washed thrice with PBS and then incubated in blocking solution for 1 h with goat anti-mouse (Invitrogen-Thermo Fisher Scientific, Milan, Italy) and goat anti-rabbit (Invitrogen-Thermo Fisher Scientific, Milan, Italy) secondary antibodies labelled with Alexa Fluor 488 and 568, respectively. To stain the nuclei, sections were incubated with DAPI in PBS for 5 min at room temperature in the dark. After three washes in PBS and a final wash in distilled water, slices were mounted using Fluoromount-G™ Mounting Medium (Thermo Fisher Scientific, Milan, Italy) as mounting medium. Digitalised images were collected at 400× total magnification using a motorized Leica microscope DM6 B equipped with a DFC9000 GT camera, supported by a THUNDER Workstation 3D DCV and by the software LAS X (Leica Biosystems, Milan, Italy). The quantitative analysis of PGP9.5- and GFAP-related immunofluorescence intensity was performed by collecting independent fields (4–6 for each animal) from the myenteric plexi and analysing them with ImageJ (NIH, Bethesda, MD, USA). The value relative to the background was subtracted from the value obtained from the analysed area, and the results were expressed as a percentage of the control group.

### 2.8. Statistics

All measurements were made by researchers blinded to animal treatments. Data were analysed using Origin 9 software (OriginLab, Northampton, MA, USA) and one- or two-way analysis of variance (ANOVA) with a Bonferroni post-test and *p* < 0.05 or 0.01 considered statistically significant. Results are shown as means ± standard error of the mean (SEM) of *n* assessments depending on the experiment.

## 3. Results

### 3.1. Characterization of Camelina Sativa Defatted Seed Meal Composition

Camelina seed meal was extracted using a mechanical food-grade process and avoiding high temperature and solvents, which could impair bioactive molecule contents and the overall safety of the new ingredient. *Camelina sativa* DSM contains 6% moisture; a relatively high protein content, 32.5% (*w*/*w* on dry matter), calculated from DSM nitrogen quantification using the conventional factor of 6.25; and a residual oil content of 24.8% (*w*/*w* on dry matter). GSL content was of 45 ± 4 µmol g^−1^, and their profile and variable side chain chemical structure are depicted in Table 1.

The residual oil of camelina DSM was mainly composed of polyunsaturated fatty acids, with linoleic (LA; 18:2,) and alpha linolenic acid (ALA; 18:3) accounting for 19% and 36.7%, respectively, plus around 15% oleic acid (18:1) and 13% gondoic acid (20:1). Erucic acid (22:1) content was low, only 2.2% of analysed fatty acids. These results agreed with literature data [39,40] and show that the defatting procedures did not drastically change the residual contents of fatty acids in the camelina DSM. The polyphenol characterization of camelina DSM was reported in Table 2 and Table 3, and the total identified soluble polyphenol fraction accounted for about 1% on a weight basis in camelina DSM. Soluble flavonoids were the most representative polyphenol fraction (77%) followed by soluble conjugated phenolic acids (16%). The insoluble fraction was mainly represented by sinapic acid (85%). Among the flavonoids, soluble conjugated flavonoids represent the majority of this class of biomolecules. The rich profile of flavonoids found in camelina DSM contains high levels of vitexin and naringenin, which account together for more than 99% of total flavonoids. Both vitexin and naringenin were considered in the recent literature for their neuroprotective effects and inhibition of inflammatory and neuropathic pain [41,42,43].

### 3.2. Acute Anti-Hyperalgesic Efficacy of Camelina Sativa DSM on Pain Associated with Colitis in Rats: Evaluation of the Role of Glucosinolates

**Figure 1:**
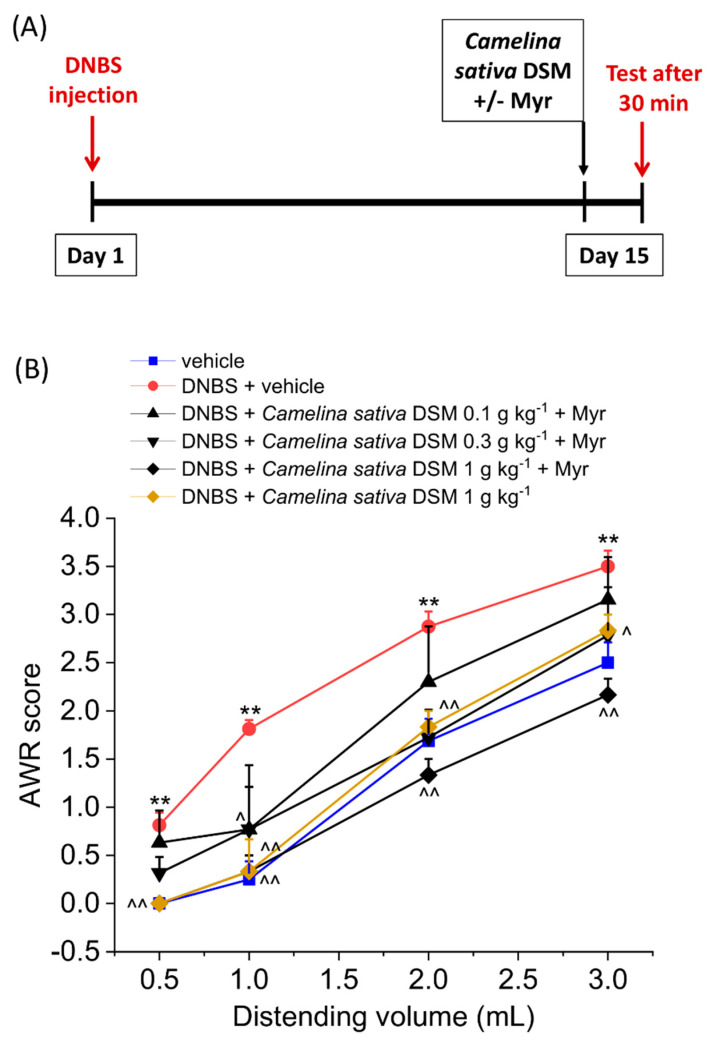
The effect of *Camelina sativa* DSM acute administration on visceral pain associated with colitis in rats before and after bio-activation with myrosinase enzyme (Myr). (**A**) Experimental scheme: the behavioural test was performed 30 min after the oral administration of the *Camelina sativa* DSM (0.1–1 g kg^−1^ p.o.). *Camelina sativa* DSM was bioactivated by adding 30 μL mL^−1^ of myrosinase (32 U mL^−1^) 15 min before the administration. (**B**) Visceral sensitivity was assessed in animals by measuring the extent of the abdominal withdrawal response (AWR) to colorectal distension, carried out by applying an increasing distending stimulus on the colon walls (0.5–3 mL). Each value represents the mean ± SEM of six animals per group. ** *p* < 0.01 vs. vehicle. ^ *p* < 0.05 and ^^ *p* < 0.01 vs. DNBS + vehicle.

**Figure 2:**
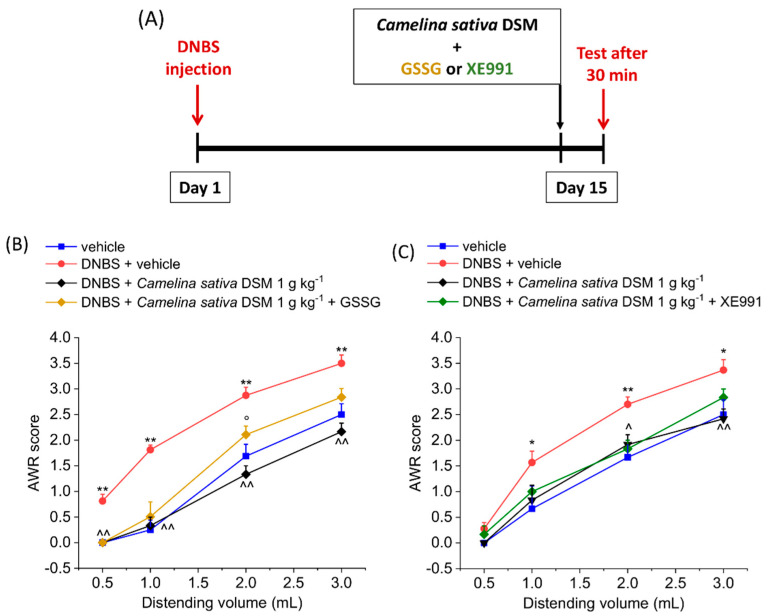
The involvement of H_2_S and Kv7 potassium channels in the acute pain-relieving effects of *Camelina sativa* DSM. (**A**) Experimental scheme: the behavioural test was performed 30 min after the oral administration of the *Camelina sativa* DSM (0.1–1 g kg^−1^ p.o.). Visceral sensitivity was assessed in animals by measuring the extent of the abdominal withdrawal response (AWR) to colorectal distension, carried out by applying an increasing distending stimulus on the colon walls (0.5–3 mL). (**B**) Oxidized glutathione (GSSG) (20 mg kg^−1^) was orally administered in concomitance with *Camelina sativa* DSM (1 g kg^−1^), and the test was performed after 30 min. (**C**) The Kv7 potassium channel blocker XE991 (1 mg kg^−1^) was intraperitoneally administered in concomitance with *Camelina sativa* DSM (1 g kg^−1^), and the test was performed after 30 min. Each value represents the mean ± SEM of six animals per group. * *p* < 0.05 and ** *p* < 0.01 vs. vehicle. ^ *p* < 0.05 and ^^ *p* < 0.01 vs. DNBS + vehicle. ° *p* < 0.05 vs. DNBS + *Camelina sativa* DSM.

The acute effect of *Camelina sativa* DSM on was evaluated in the remission phase of colitis induced by DNBS injection (Day 14; Figure 1). Visceral pain was monitored in the animals by assigning a score to their abdominal withdrawal response (AWR score; 0–4) to colorectal distension (CRD; 0.5–3 mL). Fourteen days after colitis induction, the DNBS-treated animals showed an abdominal response to CRD significantly higher in comparison with the control group receiving the vehicle (Figure 1). *Camelina sativa* DSM was bioactivated with the enzyme myrosinase (thioglucosidase, EC 3.2.1.147; Myr) before the administration in animals and was responsible for the hydrolysis of GSLs. It has been reported in previous works that bioactivation with mysrosinase can enpower the effects of GLSs *in vivo*, by increasing the availability of ITCs, which are considered to be mainly responsible for pain relief mediated by *Brassicaceae* [20]. The acute administration of the bioactivated *Camelina sativa* DSM (0.1–1 g kg^−1^ p.o.) relieved visceral hypersensitivity in DNBS-treated rats in a dose-dependent manner. Both the doses 0.3 and 1 g kg^−1^ significantly reduced the abdominal response of animals to CRD with 1–3 mL. In the group of DNBS animals receiving *Camelina sativa* DSM 1 g kg^−1^, the pain threshold was the same or even higher than in controls (vehicle treated group; Figure 1). Regarding the lower dose of the meal, although the AWR score was lower than that for the DNBS animals administered the vehicle, the effect was not statistically significant (Figure 1). The anti-hyperalgesic efficacy of *Camelina sativa* was lower when the meal was not pre-incubated with myrosinase (Figure 1). The activation of Kv7 potassium channels due to the H_2_S release *in vivo* has been described as the mechanism responsible for pain relief exerted by the GSL hydrolysis derivatives ITCs [22,24,29]. In this case, the efficacy of *Camelina sativa* DSM in relieving visceral pain in DNBS-treated animals was only slightly reduced by administering the H_2_S scavenger glutathione disulfide (GSSG) in a mixture with the meal (Figure 2A; *p* < 0.05 only with 2 mL) and was not affected by the inhibition of Kv7 potassium channels activity with the selective antagonist XE991 (1 mg kg^−1^ i.p.) (Figure 2B).

### 3.3. PPAR-α Activation Mediated the Acute Effect of Camelina Sativa DSM on Visceral Pain

**Figure 3:**
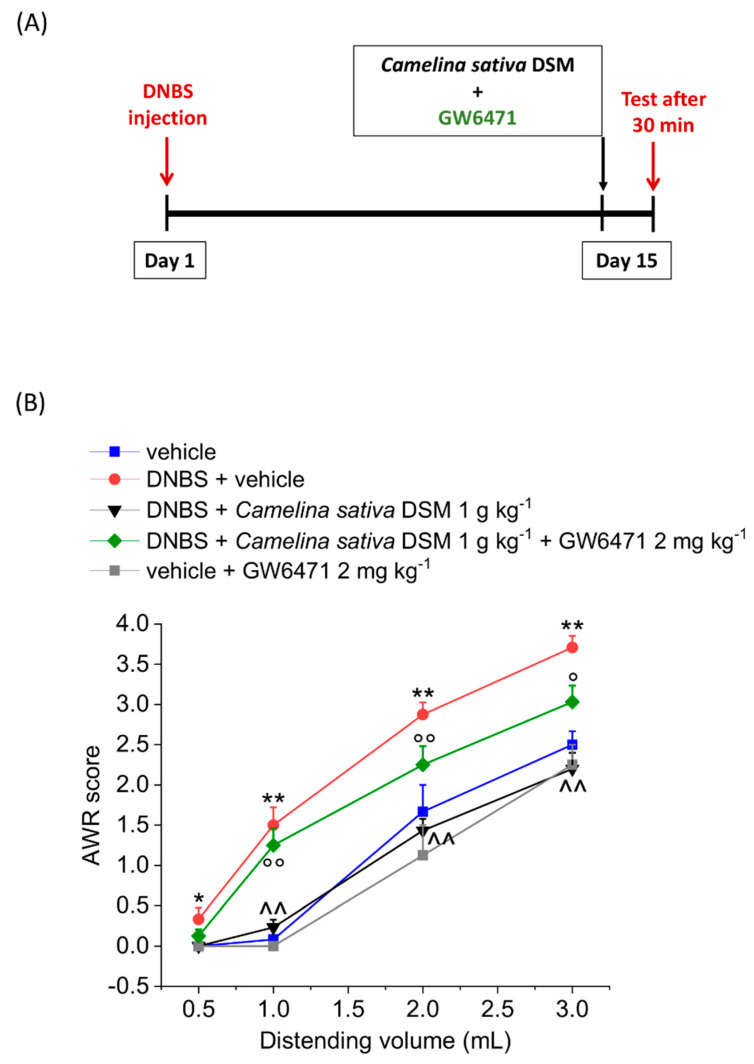
The contribution of PPAR-α receptor activation to the acute pain-relieving effect of *Camelina sativa* DSM. (**A**) Experimental scheme: the behavioural test was performed 30 min after the oral administration of the *Camelina sativa* DSM (0.1–1 g kg^−1^ p.o.). (**B**) Visceral sensitivity was assessed in animals by measuring the extent of the abdominal withdrawal response (AWR) to colorectal distension, carried out by applying an increasing distending stimulus on the colon walls (0.5–3 mL). The PPAR-α receptor antagonist GW6471 (2 mg kg^−1^) was intraperitoneally administered in concomitance with *Camelina sativa* DSM (1 g kg^−1^), and the test was performed after 30 min. Each value represents the mean ± SEM of six animals per group. * *p* < 0.05 and ** *p* < 0.01 vs. vehicle. ^^ *p* < 0.01 vs. DNBS + vehicle. ° *p* < 0.05 and °° *p* < 0.01 vs. DNBS + *Camelina sativa* DSM.

Looking for other mechanisms of action, we focused attention on the residual oil content of the defatted seed meal of *Camelina sativa*, which was still rich in polyunsaturated fatty acids with a prevalence of ALA (see Section 3.1). Among the well-known biological targets of these lipids, there are the PPAR and the cannabinoid receptors, which are both involved in pain modulation [44,45]. We observed that pre-treating the animals with GW6471 (2 mg kg^−1^), the selective antagonist of PPAR-α receptor, significantly reduced the anti-hyperalgesic efficacy of *Camelina sativa* DSM on the visceral pain caused by DNBS injection in rats, though not completely preventing its effect (Figue 3). The PPAR-α antagonist did not significantly alter the visceral pain threshold in the control group (vehicle + GW6471; Figure 3).

**Figure 4:**
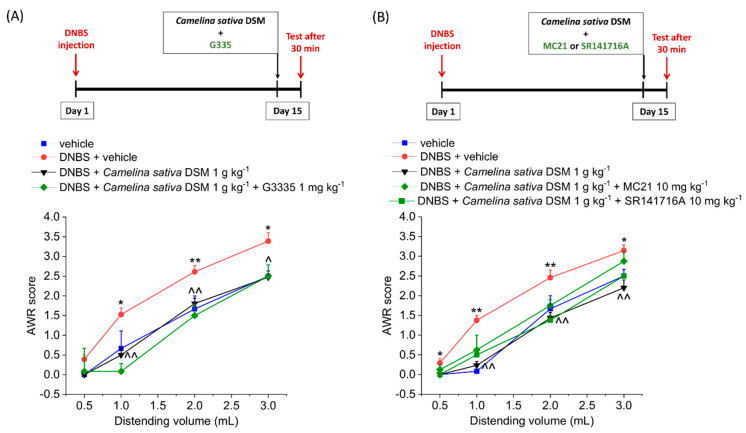
The involvement of PPAR-Ƴ, CB1, and CB2 receptor in the acute pain-relieving effect of *Camelina sativa* DSM. Visceral sensitivity was assessed in animals by measuring the extent of the abdominal withdrawal response (AWR) to colorectal distension, carried out by applying an increasing distending stimulus on the colon walls (0.5–3 mL). (**A**) The PPAR-Ƴ receptor antagonist G3335 (2 mg kg^−1^) was intraperitoneally administered in concomitance with *Camelina sativa* DSM (1 g kg^−1^), and the test was performed after 30 min. (**B**) CB1 and CB2 receptor antagonists (SR141716A and MC21, respectively; 10 mg kg^−1^) were intraperitoneally administered in concomitance with *Camelina sativa* DSM (1 g kg^−1^), and the test was performed after 30 min. Each value represents the mean ± SEM of six animals per group. * *p* < 0.05 and ** *p* < 0.01 vs. vehicle. ^ *p* < 0.05 and ^^ *p* < 0.01 vs. DNBS + vehicle.

By contrast, the efficacy of *Camelina sativa* DSM was not affected by the co-treatment with the PPAR-Ƴ (GW6471 2 mg kg^−1^ i.p.; Figure 4A), CB1 (S141716A 10 mg kg^−1^ i.p.; Figure 4B) and CB2 (MC21 mg kg^−1^ i.p.; Figure 4B) selective antagonists, respectively.

### 3.4. The Repeated Treatment with Camelina Sativa DSM Prevented the Development and Persistence of Pain Associated with Colitis in Rats

**Figure 5:**
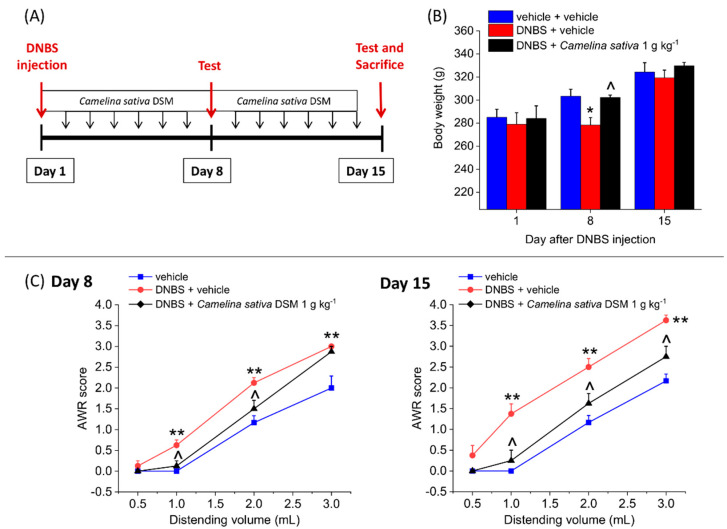
The effects of the repeated administration of *Camelina sativa* DSM in DNBS-treated rats. (**A**) Experimental scheme: *Camelina sativa* DSM (1 g kg^−1^) was administered once daily in the DNBS-treated animals, starting from the day of DNBS injection and continuing the daily treatment for 14 consecutive days. Body weight (**B**) and visceral pain threshold (**C**) were assessed on Days 8 (acute inflammatory phase) and 15 (post-inflammatory phase), 24 h after the last administration. Visceral sensitivity was assessed by measuring the extent of the abdominal withdrawal response (AWR) to colorectal distension (0.5–3 mL). Each value represents the mean ± SEM of six animals per group. * *p* < 0.05 and ** *p* < 0.01 vs. vehicle. ^ *p* < 0.05 vs. DNBS + vehicle.

We investigated the efficacy of the repeated treatment with *Camelina sativa* DSM in counteracting the development and persistence of colitis-induced visceral pain. *Camelina sativa* DSM (1 g kg^−1^) was daily administered in the animals, starting from the day of DNBS injection and continuing the daily treatment for 14 consecutive days. On Days 8 and 15, we assessed the body weight (g) of animals and their visceral pain threshold by measuring the AWR to CRD (experimental scheme; Figure 5A).

In the acute phase of colitis (Day 8 after DNBS injection), the animals displayed a significant loss of weight, which was completely prevented by the repeated treatment with *Camelina sativa* DSM (Figure 5B). Additionally, during the remission phase of colitis (Day 15), no differences were observed in the body weights among the experimental groups (Figure 5B).

DNBS-treated animals showed a higher visceral sensitivity than the controls (vehicle group) on both Day 8 and Day 15 (Figure 5 C). The abdominal response of DNBS animals to CRD was significantly lower as a result of the repeated treatment with *Camelina sativa* DSM on both Day 8 and Day 15 (Figure 5C; Day 8 *p* < 0.05 with 1–2 mL; Day 15 *p* < 0.05 with 1–3 mL), indicating that the treatment is able to counteract both the development and the persistence of colon hypersensitivity caused by colitis.

### 3.5. Camelina Sativa DSM Promoted Tissue Healing and Reduced Mast Cells Infiltration in the Colon

**Figure 6:**
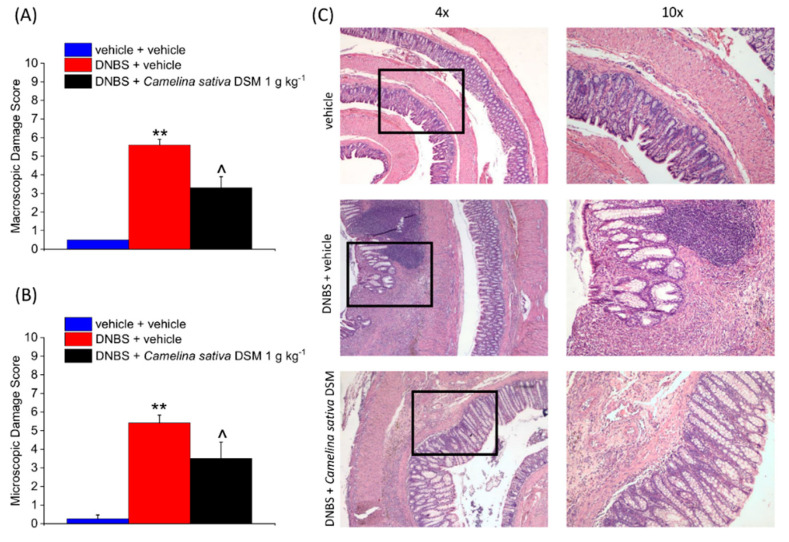
The effects of the repeated treatment with *Camelina sativa* DSM on colon damage induced by DNBS in rats. *Camelina sativa* DSM (1 g kg^−1^) was administered once daily in the DNBS-treated animals, starting from the day of DNBS injection for 14 consecutive days; then tissues were collected (Day 15). The column graphs report the colon macroscopic (**A**) and microscopic (**B**) damage score; Representative pictures of haematoxylin–eosin-stained sections of full-thickness colon (**C**). Original magnification: 4× and 10×. Each value represents the mean ± SEM of six animals per group. ** *p* < 0.01 vs. vehicle. ^ *p* < 0.05 vs. DNBS + vehicle.

On Day 15, after the behavioural test, the animals were sacrificed for evaluating the colon damage. A macroscopic damage score was assigned to the fresh tissue based on the stool consistency, the presence of hyperaemia or ulcers, and the thickness of the colonic walls (Figure 6A). In the DNBS-treated animals, the damage score was significantly higher than in the controls. The treatment with *Camelina sativa* DSM was able to reduce the damage induced by DNBS of about 40% (Figure 6A).

From a histological point of view, the colons of the DNBS-treated animals appeared thickened, with areas lacking mucosa (scars of previous ulcers) or showing irregular crypts. Moreover, a residual transmural inflammatory infiltrate can be detected in these animals (Figure 6C). The repeated treatment with *Camelina sativa* DSM reduced the intestinal damage caused by DNBS (Figure 6B). The mucosa was mostly restored, displaying regular crypts as a result of *Camelina sativa* DSM treatment. However, the inflammatory infiltrate was restricted to the submucosa in the animals receiving the meal (Figure 6C).

**Figure 7:**
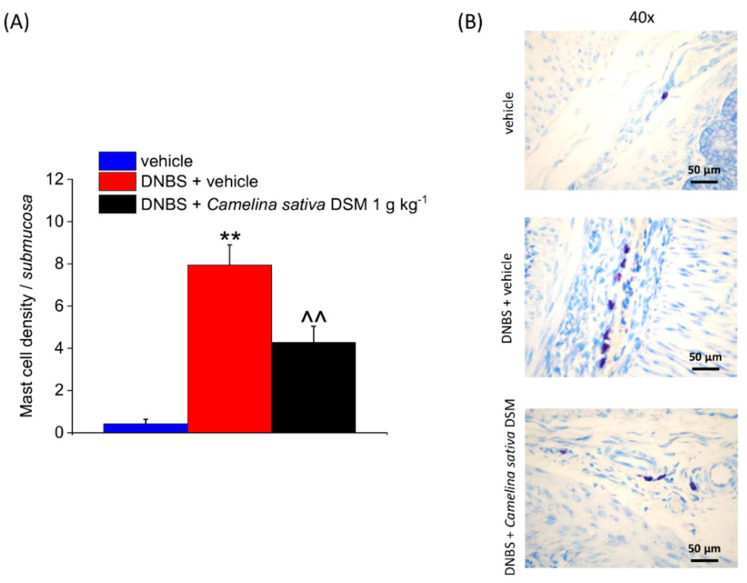
The effects of the repeated treatment with *Camelina sativa* DSM on submucosal mast cell infiltration caused by DNBS. *Camelina sativa* DSM (1 g kg^−1^) was administered once daily in the DNBS-treated animals, starting from the day of DNBS injection for 14 consecutive days, and then tissues were collected. The column graph displays the mean mast cell density per area of colonic wall (cells/field) (**A**). The panel shows pictures captured from submucosa of mast cell granules stained in purple with GIEMSA (**B**). Each value represents the mean ± SEM of six animals per group. ** *p* < 0.01 vs. vehicle. ^^ *p* < 0.01 vs. DNBS. Original magnification: 40×.

Visceral pain correlates with an overactivity of mast cells within the colons of both animals and humans [8,46,47,48]. The histological analysis confirmed a persistent increase in mast cell density within the submucosa of the DNBS-injected animals (Figure 7A). *Camelina sativa* DSM treatment lowered the number of mast cells infiltrating the submucosa after colitis induction by about 50% (Figure 7A). The differences observed among the experimental groups clearly emerge in the GIEMSA-stained mast cells’ granules in Figure 7B.

### 3.6. Neuroprotective Effect of Camelina Sativa DSM on the Colon

**Figure 8:**
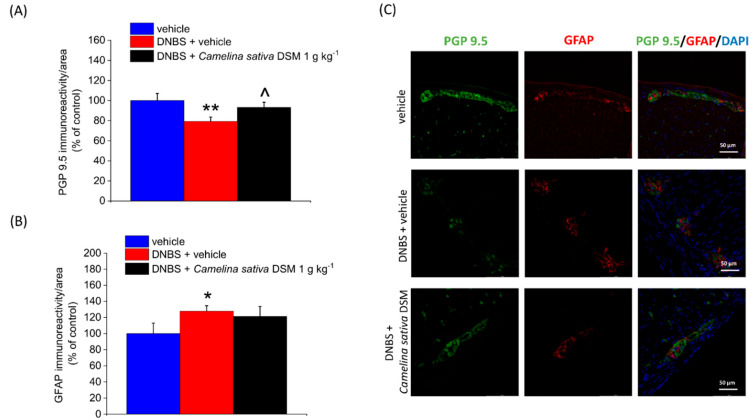
The neuroprotective effects of *Camelina sativa* DSM on the colonic myenteric plexus of DNBS-treated rats. *Camelina sativa* DSM (1 g kg^−1^) was administered once daily in the DNBS-treated animals, starting from the day of DNBS injection for 14 consecutive days, and then tissues were collected. The immunolabeling quantification of PGP 9.5 (**A**) and GFAP (**B**) with relative immunofluorescence images showing the expression of PGP 9.5 (green), GFAP (red), and DAPI (blue) in the myenteric plexus of the colon (**C**). The quantitative analysis of PGP9.5- and GFAP-related immunofluorescence intensity (arbitrary unit) was performed by collecting independent fields (4–6 for each animal) from the myenteric plexi. Results were expressed as a percentage of the control group (vehicle-treated animals). Each value represents the mean ± SEM of six animals per group. * *p* < 0.05 vs. vehicle. ** *p* < 0.01 vs. vehicle. ^ *p* < 0.05 vs. DNBS + vehicle. Original magnification: 40×.

The damage to enteric neurons, as well as the remodelling of enteric neuronal networks, contributes to the development of post-inflammatory visceral hypersensitivity in animals [8,49]. The damage to enteric neurons caused by intestinal inflammation is attested to by the slight but significant reduction in the immunoreactivity of the neuronal marker PGP 9.5 observed in the colon myenteric plexus of DNBS-treated animals (Figure 8A).

Moreover, during inflammatory conditions, enteric glia surrounding neurons in the gut can undergo extensive changes which alter the interaction with the heterogenous cellular environment, contributing to perpetuating visceral hyperalgesia [50,51,52,53]. Enteric glia activation can be highlighted in animals and humans by changes in the expression of peculiar biological markers, such as GFAP [52,53,54]. In Figure 8B, an up-regulation of GFAP expression in DNBS-treated rats with respect to controls (vehicle) can be observed.

The repeated treatment with *Camelina sativa* DSM (1 g kg^−1^) significantly protected enteric neurons from the inflammatory damage (Figure 8A, illustrative images in Figure 8C) but did not prevent the activation of enteric glia caused by DNBS (Figure 8B, illustrative images in Figure 8C).

## 4. Discussion

*Camelina sativa* DSM effectively counteracted the visceral hyperalgesia associated with colitis in rats. The activation of the PPAR-α pathway contributed to the acute anti-hyperalgesic efficacy of the meal. *Camelina sativa* DSM also prevented the development and the persistence of post-inflammatory pain by a combination of mechanisms, including the promotion of tissue healing, the reduction of mast cell infiltration, and the protection of enteric neurons from the inflammatory insult.

Gut inflammation represents a major risk factor for developing visceral hypersensitivity, which characteristically persists even after the resolution of intestinal damage [1,12,55]. To date, many plant-derived products have been studied with the aim of combining different mechanisms of action to improve the therapy for post-inflammatory intestinal hyperalgesia, which is supported by inflammatory, neuropathic, and immune mechanisms [1,8]. In this context, *Brassicaceae* plants emerged to be ideal candidates, since the ITCs contained in these plants can modulate both inflammatory response and oxidative stress [9,10]; they also display potential benefits for the gut microbiota [12,13,14,56], which has high relevance in gut pain associated with colitis [17,57,58]. Several studies in the literature attest that ITCs are effective against both inflammatory and neuropathic pain by the slow release of H_2_S *in vivo*, and by the positive modulation of Kv7 potassium channels activity [18,20,21,25,59], which are involved in different physiological processes like the regulation of blood pressure and the transmission of painful stimuli [11,19,60,61,62].

*Camelina sativa* is an ancient oil crop belonging to the *Brassicaceae* family. The seed of this plant has a high content of *n*-3 fatty acids and recently elicited great interest for the production of functional food [26,27,28]. Additionally, the defatted seeds of camelina are rich in GSLs like glucoarabin and glucocamelinin [29,30,31], although the biological effects of these phytochemicals are still largely unknown. There are several studies in the literature reporting the therapeutic potential of *Camelina sativa* oil in metabolic disorders [55,63], whereas there is little evidence about other effects, such as the regulation of the immune response and the inflammatory processes [29,55,57,64].

As was observed in patients, in the rat model of colitis induced by the intrarectal injection of the sensitizing agent DNBS, the intestinal damage is accompanied by the development of persistent visceral hypersensitivity [8]. The acute administration of *Camelina sativa* dose-dependently reduced gut hyperalgesia in these rats. This effect was enhanced by the bioactivation with myrosinase, which increases ITCs availability, suggesting their involvement in camelina-mediated pain relief. However, co-administering glutathione, able to prevent the release of H_2_S from the ITCs moiety, only partially reduced the pain-relieving efficacy of the meal, while the Kv7 blocker XE991 did not show the same effect. Taken together, these findings suggest that ITCs are only partially responsible for the anti-hyperalgesic efficacy of *Camelina sativa* DSM irrespective of the activation of the Kv7 channels.

Looking for the mechanism underlying camelina-mediated pain relief, we shifted attention to the residual oil content of the defatted seed meal, which is rich in polyunsaturated fatty acids. Several lipids were found to be endowed with analgesic or anti-hyperalgesic effects, among them endocannabinoids such as anandamide and 2-arachidonoyl-*sn*-glycerol; lipid-amide agonists of PPAR-α such as palmitoylethanolamide and oleoylethanolamide; and various products of the oxidative metabolism of polyunsaturated fatty acids [58]. These lipid mediators are differently released during the course of inflammation, and they have been proposed as the peripheral actors that regulate the access of nociceptive information to the central nervous system [58]. Moreover, lipid mediators derived from the ω-3 polyunsaturated fatty acid precursors docosahexaenoic acid and eicosapentaenoic acid have been found to inhibit thermal and mechanical hypersensitivity in preclinical models of somatic and visceral inflammatory pain [65,66]. We observed that pre-treating the animals with the selective PPAR-α receptor antagonist GW6471 almost completely prevented the acute pain-relief offered by camelina administration in DNBS animals. The effect was independent of the activation of PPAR-Ƴ receptor or the CB1 and CB2 receptors, which are involved in pain regulation as well [43,49,50,67,68]. Interestingly, PPAR-α is involved in the modulation of pain signalling [69], as well as in several pathophysiological mechanisms, some of which contribute to pain development such as neuroinflammation and oxidative stress [70].

We observed that the repeated treatment with camelina was effective in counteracting the development of visceral hyperalgesia after the acute intestinal inflammation, as well as in preventing its persistence in the remission phase of colitis induced by DNBS. *Camelina sativa* DSM also prevented the body weight loss caused by DNBS injection in rats. Although the caloric intake of the meal was not investigated, in the remission phase of colitis, we found no significant differences in the body weights of animals treated with *Camelina sativa* DSM with respect to both DNBS and control animals receiving the vehicle. This suggests that the animals felt better, which is in line with the behavioural data. At the same time, the treatment with camelina was able to partially reduce the intestinal damage caused by colitis and strongly lower the number of mast cells infiltrating the tissue. This last parameter has high relevance in the context of visceral pain, which has been linked to a deregulation of mast cell function [52,53,71,72]. The control of mast cell activity is also important in the maintenance of gut homeostasis since these cells are the crossroads of many physiological processes playing a key role in the regulation of the intestinal barrier, secretion, and gut motility [48,73].

Inflammatory processes also cause damage to myenteric neurons as attested to by the slight but significant reduction in the expression of the neuronal marker PGP 9.5, as previously reported [49]. The damage to neurons, together with the overactivation of enteric glial cells, are thought to be responsible for the altered enteric nervous signalling observed in patients affected by chronic visceral hypersensitivity [59,60,74,75]. The repeated treatment with *Camelina sativa* DSM significantly counteracted the damage to enteric neurons caused by DNBS but did not prevent the activation of enteric glia induced by DNBS, suggesting a possible direct protective effect of camelina on neurons. The hypothesis of a direct neuroprotective action of camelina constituents is supported by the fact that the treatment strongly reduced visceral pain in the animals, even if the colon was not completely healed and the glial cells still displayed enhanced activity. Nevertheless, further studies are needed to elucidate the mechanisms behind the wide range of beneficial effects showed by camelina in the rat model of colitis.

Like the pain-relieving effect, the neuroprotective effect of camelina might be mediated by the activation of PPAR-α receptors, even if we cannot exclude other mechanisms. Literature evidence attested to the anti-inflammatory and neuroprotective effects of PPAR receptor agonists like fenofibrate in different pathologies [67,76,77,78]. Interestingly, in neuropathic conditions like sciatic nerve injury, pain relief and neuroprotection share a PPAR-α-mediated mechanism [45,79]. The genetic ablation of PPAR-α has been reported to affect both neuropathic and visceral nociception. Notably, the altered pain signalling observed in PPAR-α null mice seems to result from biological adaptations to receptor deletion because blockade of PPAR-α does not affect inflammatory pain or thermal reactions [80]. The last finding is in line with our evidence showing no effect of the PPAR-α antagonist on the controls’ visceral pain thresholds.

Anyway, it is important to take in account that other phytochemicals, like the GSLs and the flavonoids, have a neuroprotective potential [9,18] that might involve PPAR-α and/or be completely independent from the modulation of these receptors. Interestingly, flavonoids such as naringenin can modulate the activation of PPAR-α [68,81,82], while GSLs and ITCs are reported to act on the signalling pathways modulated by PPAR-α, such as related to NF-kB [9,71,72]. This convergence might potentiate the effect of *Camelina sativa*’s constituents on the intestinal damage. It has been also shown that the flavonoid components of *Brassicaceae* can enhance skin barrier function by positively modulating PPAR-α receptor and suppressing inflammatory cytokine production [74], a mechanism which might strengthen the gut barrier. *Camelina sativa* DSM has a high content of vitexin and naringenin. These flavonoids are endowed with a neuroprotective potential, attested to by their ability to counteract inflammatory and neuropathic pain [42,43,44]. Behind the anti-inflammatory effects, vitexin-enriched plants exhibited remarkable pain-relieving activities. Investigations of the anti-nociceptive properties of vitexin shed light on the multitarget nature of this flavonoid, which can modulate different receptors (opioid, GABA_A_, TRPV1) and processes (oxidative stress, cytokine production) involved in pain [42,75,83]. Additionally, naringenin might support the anti-oxidative and anti-inflammatory effects of *Camelina sativa* DSM on the gut [84]. Indeed, it is possible that the camelina meal, by alleviating inflammation and oxidative stress, indirectly prevents neuronal damage. In this regard, it is also important to consider that defatted seed meals contain a good amount of fibers and that those of *Camelina sativa* are known for being particularly rich in mucilage, crude fibres, and lignin, which could provide additional benefits for intestinal health [85], either alone or in combination with other bioactive molecules. The relevance of fiber content in *Camelina sativa* DSM deserves further dedicated analysis, including of its effects on the microbiome, which is a key partner in the regulation of visceral sensitivity [15].

Finally, it is interesting to note that methanolic and ethanolic extract of *Camelina sativa* were able to alleviate oxidative stress, memory deficits, and affective impairments in a mouse model of irritable bowel syndrome caused by stress exposure [32]. Altogether, these findings suggest the possibility of a synergic effect of *Camelina sativa* constituents on visceral pain and comorbidities associated with colitis. Future research aimed at further investigating the effects exerted by the active constituents of *Camelina sativa* seeds is warranted and will help to optimize the efficacy of the meal and the translatability to human therapies.

## 5. Conclusions

This work has shed light on new mechanisms underpinning the acute anti-nociceptive effects of *Camelina sativa*, such as PPAR-α activity modulation. It is likely that the components of *Camelina sativa* seeds, including GSLs, fatty acids, and flavonoids, exert synergic effects, promoting gut health by different mechanisms. Therefore, *Camelina sativa* DSM might represent a multitarget approach to effectively managing persistent symptoms, such as hypersensitivity, and restoring of tissue homeostasis in patients affected by intestinal diseases, as a food supplement or alternatively as a functional food. Indeed, the combination of the efficacy and the safety showed by camelina encourages the long-term employment of this preparation for the treatment of persistent visceral pain.

## Figures and Tables

**Table 1:** Glucosinolates in *Camelina sativa* DSM. The contents are expressed in μmol g^−1^ of DSM. Means ± standard deviation (*n* = 3) are shown. Glucosinolate common variable side chain (R) chemical structure is also indicated, where X represents the GSL S-glucopyranosyl thiohydroximate moiety.

Glucosinolate	Side Chain (R) Structure	Content (μmol g^−1^)
Glucoarabin	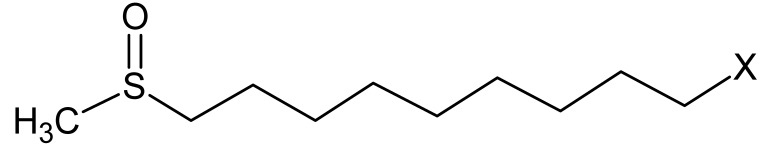	10.9 ± 0.9
Glucocamelinin	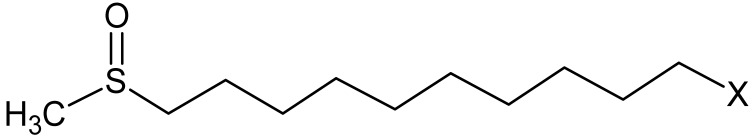	29 ± 2
11-methylsulfinylundecyl Glucosinolate	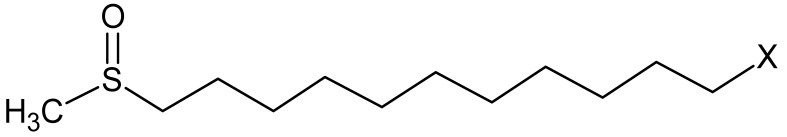	5.0 ± 0.7

**Table 2:** Soluble conjugated and insoluble bound phenolic acids and aldehyde (μg g^−1^) in *Camelina sativa* defatted seed meal (DSM). Average values ± standard deviation (*n* = 3) are shown.

	Soluble Conjugated Phenolic Acids and Aldehyde	Insoluble Conjugated Phenolic Acids and Aldehyde
Gallic acid	273 ± 27	17.4 ± 4
Protocatechuic acid	4.4 ± 0.5	**n**.d.
*p*-hydroxybenzoic acid	443.1 ± 0.8	n.d.
Vanillic acid	29.7 ± 0.2	n.d.
Syringic acid	13.2 ± 0.2	1.8 ± 0.3
Vanillin	44 ± 3	3.1 ± 0.3
Caffeic acid	9.8 ± 0.1	8.5 ± 1.7
*p*-coumaric acid	177 ± 16	34 ± 6
Sinapic acid	461 ± 25	758 ± 78
*trans*-cinnamic acid	853.2 ± 0.4	50.1 ± 1.4

n.d. not detected.

**Table 3:** Soluble and insoluble flavonoids (μg g^−1^) in *Camelina sativa* defatted seed meal (DSM). Average values ± standard deviations (*n* = 3) are shown.

	Soluble Conjugated Flavonoids	Insoluble Conjugated Flavonoids
Luteolin	287 ± 40	n.d.
Vitexin	7486 ± 46	1.8 ± 0.3
Apigenin	473 ± 13	10 ± 3
Naringenin	2038 ± 46	n.d.
Rutin	302 ± 22	6.0 ± 0.1
Quercetin	209 ± 1	n.d.

n.d. not detected.

## Data Availability

The data presented in this study are available on request from the corresponding author. The data are not publicly available due to privacy restrictions.
